# Construction and validation of the quality of oncology nursing care scale (QONCS)

**DOI:** 10.1186/s12912-014-0048-4

**Published:** 2014-12-19

**Authors:** Andreas Charalambous, Theodoula Adamakidou

**Affiliations:** Cyprus University of Technology, 15th Vragadinou Street, 3041 Limassol, Cyprus; Department of Nursing Technological Educational Institute (TEI) of Athens, Athens, Greece; University of Turku, Turku, Finland

**Keywords:** Validation, Quality nursing care, Cancer, Oncology setting

## Abstract

**Background:**

There is scarcity of questionnaires specifically on the quality of the nursing care provided to patients diagnosed with cancer. The available questionnaires have been developed without attributing a holistic approach to the care provided with important patient’s needs remaining without assessment. The main aim was to develop a self-administered cancer specific questionnaire exploring patients’ views on quality nursing care provided in oncology settings.

**Methods:**

The development of the scale proceeded through three phases. As part of the first development phase areas of concern and items of interest were identified through a literature review. The second phase included a pilot study of the QONCS and a subsequent validation phase through a multicentre study in 3 hospitals, 4 departments and 418 patients diagnosed with cancer and receiving care as inpatients. The study was designed to select items, identify dimensions, measure reliability, content and construct validity.

**Results:**

The QONCS consisted of 34 items. A factorial analysis grouped the items into five categories that define quality nursing care: a) Being supported and confirmed, b) Spiritual caring c) Sense of belonging, d) Being valued and e) Being respected. Cronbach’s alpha was 0.95 for the entire questionnaire. The factor solution explained 68.53% of the variance.

**Conclusions:**

QONCS appears to measure with adequate reliability and validity the attributes of quality nursing care within the oncological settings and to patients with a variety of cancer diagnoses and at different phases of the cancer trajectory. The instrument is quick to disseminate and easy to complete, making it a suitable instrument for nursing professionals to evaluate patients’ self-perceived quality of nursing care as a mean to promote the quality of the care provided in oncological settings.

## Background

Healthcare providers have embarked on a longstanding task to provide the best possible care to patients diagnosed with various types of cancer. The contribution of the nurses has been acknowledged throughout the earlier times when the aim was to attain quality care to the later ones where the focus was shifted to retaining the high levels of care [1]. Furthermore, their contribution extended to the contemporary aim to promote and personalise care [1,2]. Nursing’s influence on the provision of quality care was identified in the literature [3] and one that was attributed to various reasons. These include the time the nurse spends with the patient [4], the intimate relationship developed between the patient and the nurse [5] the high levels of trust characterizing this relationship [6] and the good communication [7].

Patients diagnosed with and on treatment for cancer face multiple health related problems and explicit needs that call for a complex and individualised care. This complexity within the cancer care setting requires a multidisciplinary (MDT) collaboration for the comprehensive support of the patient and the family. Within this multidisciplinary framework, clinical nurse specialist and advanced nurse specialist [8] identify and address different physio-psycho-social needs of patients by employing a holistic approach in cancer care [9]. This is informed by the philosophy of nursing that calls for nurses to undertake advanced assessment of the care in order to identify any gaps that negatively reflect on the perceived level of care received [9]. In order to achieve a holistic assessment of the patient’s needs and to promote patient-centred care, nurses draw on advance communication skills that play a crucial role in bridging the communication gap between the service provider and the patient [10,11]. Their unique position in proximity to patients enables the nurses to assume the role of MDT coordinators [10]. This role allows them to identify patients for discussion prior to the MDT meeting, organising meetings, and coordinating the logistics for the MDT meeting [12,13].

The complexity of the care [9,14] along with the difficulties in establishing a consensus definition on quality nursing care [1] were reflected in the limited number of disease-specific questionnaires available to measure the quality levels of the nursing care. Only the Oncology Patients’ Perceptions of the Quality of Nursing Care Scale (OPPQNCS) was developed specifically to evaluate the nursing care in the oncological setting [15]. These relevant questionnaires present with a number of theoretical and conceptual limitations that confine their ability to assess nursing care. These limitations include the lack of a clear quality nursing care definition and a theoretical framework that informed their development such as the Quality Patient Care Scale (QUALPACS) [16] and the Palliative Care Quality of Life Instrument (PQLI) [17]. Furthermore some of the available questionnaires were not based on patients’ perceptions theoretically limiting their validity as in the case of the Rush Medicus Tool- Monitoring the Quality of Nursing Care [18]. As was discussed earlier caring for the patient holistically is of essence for quality nursing care, however preceding questionnaires failed to assess the care in a holistic manner. Therefore the spiritual aspects of the care are not adequately addressed by the existing questionnaires such as in the case of the OPPQNCS [15]. The use of these questionnaires was further limited by several factors, primarily the stage of cancer and the active treatment. Furthermore, within the healthcare arena the published questionnaires are used to evaluate self-perceived overall quality of the care with a lack of emphasis on the nursing care specifically. Moreover the lack of focus on the needs deriving from the cancer or its treatment is also apparent [1]. Despite their limitations these questionnaires can help to identify individual patients and groups who merit additional attention or even targeted interventions. These can also highlight areas of the care process that can be improved or identify situations where there is need for other specialised healthcare professionals to be involved in the care. Furthermore, these scales can be used as tools to improve the cancer care experience for the patient and the family.

The rationale to the development of the new scale was to address the limitations of the preceding questionnaires. The outcome of the development and validation processes will be a scale to assess the self-perceived quality levels of the nursing care from a holistic perspective. This scale will be appropriate for use in adult patients diagnosed with various types of cancer and on active treatment.

One of the major limitations of the preceding questionnaires was the lack of a theoretical or conceptual framework that guided their development phase. Therefore, a hermeneutic phenomenological exploration of the concept of quality nursing care as this was perceived by cancer patients, their advocates and nurses preceded the development and validation processes [1]. The aim of this qualitative work was to establish a conceptual framework that would informed the development of the new scale. The data for this qualitative work were collected with narratives. One open-ended question was used, inviting the participants to narrate their experiences in relation to the topic under investigation. Patients narrated their stories about being cared for by the nurses in the oncology departments. The researchers aimed to explore the experiences of nurses providing care to cancer patients in these departments. Therefore they invited nurses to narrate their experiences of providing care to patients in light of what they perceived as quality nursing care. Patients’ advocates narrated from their point of view what they perceived as quality nursing care by recalling relevant experiences. Through the narratives the researchers extracted words and expressions that could be useful for creating a conceptual framework of quality nursing care. Furthermore, through the findings it was highlighted that cancer care can be complicated and it demands a holistic approach to assessing and addressing the needs of the patient and the family. Within this holistic paradigm patients found the attention attributed by nurses to their spiritual needs lacking causing a negative impact on their perceived quality of nursing care. The results demonstrated how the little and simple things in the care of the patients are the ones that count to and appreciated the most by the patient. It is seemingly simple acts, such as being there, holding a patient’s hand and listening that can bring such profound changes in comfort when one is ill [1].

## Methods

The authors employed a combination of methods for the development and validation of the QONCS. The development and validation proceeded through three phases. The first phase included the identification of items for the questionnaire and the second phase involved the pilot testing of the first version of the instrument. The final phase comprised of a multi-centre descriptive study designed to psychometrically evaluate the questionnaire by administering it to the target population, adult inpatients diagnosed with cancer. International guidelines and methodologies informed the development process of the QONCS [19].

### Item development and description

The authors performed a review of the literature by searching for papers and questionnaires related to the quality of nursing care. PubMed, CINAHL, and EMBASE scientific databases were consulted looking for research published between 1974 and 2010. The following search terms were utilised: “quality” AND “care” AND “nurs*” AND “scale” OR “test” OR “instrument” AND “measure*” OR “psychometr*” AND “inpatient” AND “cancer” OR “oncology*”. The search was limited to English. Types of papers selected were those describing instruments that measure the quality of the provided nursing care within the hospital setting. Reference lists of articles investigating this topic were also examined for relevant studies. The searching period began in February 2009 and ended in June 2009. Finally, 29 original questionnaires were reviewed. A thorough analysis was also performed to identify examples of items. Table 1 shows the most relevant articles reviewed for the items generation.

**Table 1:** **Characteristics of questionnaire/scale reviewed for items identification**

**Author**	**Questionnaire/scale**	**Item categories**	**Population**
Radwin, Alster and Rubin, 2003 [15]	Oncology Patients Perceptions of the Quality of Nursing Care Scale (OPPQNCS)	● Responsiveness	Adult In-patients
		● Individualization	
		● Coordination	
		● Proficiency	
Wandelt and Ager, 1974 [16]	Quality Patient Care Scale (QUALPACS)	● Psychosocial-individual	Adult In-patients
		● Psychososial-group	
		● Physical	
		● General	
		● Communication	
		● Professional implications	
Haussmann,Hegyvary, Newman and Bishop, 1974 [18]	Rush-Medicus Quality Monitoring Instrument (RMT-MQNC)	● Plan of nursing care is formulated	Adult In-patients
		● Physical needs of the patients are met	
		● Psychological, social, emotional and mental needs of the patient are met	
		● Achievement of nursing care objectives is evaluated	
		● Procedures are followed for protection of all patients	
		● Delivery of nursing care is facilitated by administration and managerial services	
Lynn, McMillen and Sidani, 2007 [32]	Patient’s Assessment of Quality Scale Acute Care Version (PAQS-ACV)	● Individualization	Adult In-patients
		● Nurse characteristics	
		● Caring	
		● Environment	
		● Responsiveness	
Mystakidou et al. 2004 [17]	The Palliative Care Quality of Life Instrument (PQLI)	● Activity	Adult In-patients
		● Self-care	
		● Symptom scale	
		● Choice of treatment	
		● Psychological Affect	
		● Overall quality of life	
Lee, Hsu, Chang, 2007 [33]	Orthopedic Nursing Care Quality Monitor Tool	● Plan of nursing care is formulated	Adult In-patients
		● Physical needs of the patients are met	
		● Psycho-social-spiritual needs of the patient were attended	
		● Achievement of nursing care objectives was evaluated	
Risser, 1975 [30]	Risser Patient Satisfaction Scale (RPSS)	● Technical–professional,	Adult Out-patients
		● Interpersonal educational	
		● Interpersonal–trusting	
La Monica, Oberst., Madea, Wolf 1986 [29]	La Monica – Oberst Patient Satisfaction Scale (LOPSS)	● Dissatisfaction,	Adult In-patients
		● Interpersonal support	
		● Good impression	
Laschinger, H.K.S., McGillis Hall, L., Pedersen, C., & Almost, 2005 [31]	PSNCQQ - Patient Satisfaction with Nursing Care Quality Questionnaire	● Assessment of individualization of care,	Adult In-patients
		● Concern and caring by nurses,	
		● Skill and competence of nurses,	
		● Collaboration among nursing staff,	
		● Provision of comfort,	
		● Responsiveness of nurses,	
		● Information provided by nurses,	
		● Discharge instructions and	
		● Coordination of care after discharge	
Marife Mamauag Carlo Magno, 2005 [34]	NQS scale – Nursing Quality Scale	● Caring	Nursing Students
		● Compassion	
		● Commitment	
		● Connectness	

Drawing on the conceptual framework of quality nursing care and the literature review, a pool of statements was prepared to achieve an adequate sample of items within each of the six major content areas that comprise the quality nursing care construct as this was described by Charalambous et al. [1,20]: being valued, being respected, being cared for by communicative and supportive nurses, being confirmed, being cared for religiously and spiritually and sense of belonging. The items were re-phrased into affirmative statements and asked the informants to rate their self perception of quality nursing care using a 5-point Likert-type scale, where 1 denoted “Strongly disagree” to 5 that denoted “Strongly agree”. A pool of 85 potential items formed the first version of the scale.

The selection of the items was carried out in two stages. In the first stage eleven nurse experts were asked to classify the items into one of six dimensions that comprised the quality nursing care construct. Items were included in one of the six dimensions if an agreement level of over 75% was achieved between the experts. Ambiguous items that could not clearly be classified in one of the dimensions were excluded by the experts. For example, the item “Generally I feel that I am in uncertain hands” was removed from the final version of the questionnaire. A total of 23 items were eliminated, mainly from the “being confirmed” and “being cared for religiously and spiritually” dimensions. Once this process was completed the experts were asked to provide evaluations in relation to the level of relevance of each item for its corresponding dimension of quality nursing care. The items were classified according to three categories: 3 “essential”, 2 “interesting but not essential” and 1 “irrelevant”. For each item the CVR (content validity ratio) was calculated [21] as well as the criterion content validity index (CVI) for the entire scale. Only the items that met the CVR of > or = .73 were retained in the scale. The following version of the questionnaire was reduced to 45 items. Fifteen inpatients were selected in order to assess the comprehension (how items were interpreted) and feasibility of the reviewed pool of items and format response. Participants were selected based on explicit inclusion and exclusion criteria. Patients were included if they had a histopathological diagnosis of cancer, were 18 years or older, on active treatment and were receiving care at the hospital as inpatients. Eligible participants needed to be able to speak and understand Greek and they had given written informed consent. Participants should also have a score of >50 on the Karnofsky Performance Scale Index [22] and a mean of >50 on the Attentional Function Index (AFI) [23]. Patients were excluded if they were receiving palliative care or they had an impaired cognitive ability.

The second phase and after modifying the items according to the inpatients recommendations the first draft of the QONCS (45 items) was administered to a convenience sample of 100 patients diagnosed with various types of cancer and receiving care at 3 hospitals in Cyprus. The aims of this phase were to evaluate the quality of generated items and eliminate those proving to be inadequate.

### Description of the questionnaire

To collect more information and to use it for further analyses the scale was designed with 2 sections. The first section asked the respondent to describe their personal characteristics, including age, gender, area of residence, family status and educational level as well as information regarding cancer and its treatment including type of cancer, time of diagnosis, type of treatment, days of treatment and whether they were receiving care for the first time. The next section was the QONCS. The “being valued” subscale asked the respondents to express the level of agreement over five questions referring to the value attributed to them by the nurse during the care. The “being respected” subscale included eight questions that related to the level of respect the patients received during their care by the nurse. The “being cared for by communicative and supportive nurses” subscale included four questions referring to the quality of communication and the level of support offered by the nurse during the care. The next subscale, “being confirmed”, included six questions seeking to assess the sense of safety and trust generated by the nurse. The “spiritual caring” subscale was comprised of six questions referring to existential issues raised by the patients and whether the nurse met these needs. Finally the “sense of belonging” subscale assessed family issues raised during the care of the patient through five questions.

In phase three, this version of the questionnaire was administered to inpatients diagnosed with cancer between June of 2010 to October 2010. The respondents completed the questionnaire on two distinct times. The first assessment took place on the second day of their admission and the second 4 days later. The study was undertaken simultaneously in 2 hospitals in Cyprus and one hospital in Greece. The convenient sample consisted of adult patients diagnosed with various types of cancer receiving inpatient care.

### Ethical considerations

The study complied with all the principles of the Helsinki Declaration [24] as these were developed by the World Medical Association (WMA). Approval was obtained by the Bioethics Committee in Cyprus and the Ethics Committee at St. Savvas Anticancer Hospital in Greece. A participant information sheet providing details of the study accompanied the questionnaire. Written and oral consent was assured by all the informants at each phase of the study. Patients were informed that their participation in the study was strictly on a voluntary basis and in the event they wished to decline their participation in the study, there would be no consequences in relation to their treatment, hospitalization and care.

### Data analysis

Cronbach’s alpha was used to quantify the internal reliability of the total questionnaire as well as the 6 factors, and to assess the contribution of each question to the overall reliability of each factor. Content validity was addressed through the rigorous process employed to develop the instrument. This process included basing the items on the prior instruments. Furthermore, the process was fortified by the collegial development of the new items using a panel experienced in quality issues pertaining the nursing care provided to patients diagnosed with cancer. Stability (test-retest) reliability was determined with Spearman’s correlation coefficients between the first (time 1 = test) and second (time 2 = retest) responses of the scale’s items.

Data were analysed using IBM SPSS version 20. Frequencies were used to describe the characteristics of the sample.

Construct validity was carried out using pairwise deletion of missing values and conducting a factor analysis with the Maximum Likelihood method with Oblimin rotation for each item set. The sorted factor loadings, eigenvalues and scree plots resulting from these analyses were examined to identify the number of dimensions or factors that made up the best solution for each item set. The factor loadings were examined to determine whether all items in the set were associated with the attribute of interest [25].

## Results

### Description of the sample

The process of item development and selection has been described in the Methods section. The results reported here refer to the pilot administration and the final validation of the QONCS to the target population. The sample population of patients diagnosed with cancer and receive treatment in the hospital (n = 100) completed a pilot administration of the 45-item QONCS. Cronbach’s alpha coefficient for the first administration was 0.80. Through an item analysis we discarded those items that were either highly correlated with other items, and were thus considered repetitive, or that had item-scale correlations less than 0.30. In total 11 items had these characteristics and were excluded from the final version of the scale to assess quality nursing care in the oncological setting that consisted of 34 items.

The final version of the QONCS was subjected to test-retest reliability testing with an overall response rate of 76.9% (n = 418). The majority of the patients that were included in the study were male (54.3% [227] vs. 45.7% [191]). One hundred and seventy six patients (42.8%) were diagnosed with cancer within a period of less than a month whilst 172 patients received their diagnosis over 2 months (41.1%). The detail description of the sample’s characteristics appears in Table 2.

**Table 2:** **Sample’s characteristics**

**Variable**	**Frequency**	**Percentage (%)**
**Gender**		
Male	227	54.3
Female	191	45.7
**Area of residence**		
Urban	367	87.8
Rural	51	12.2
**Age**		
18-28	32	7.6
29-39	33	7.8
40-50	66	15.8
51-60	78	18.7
61-70	122	29.3
>70	87	20.8
**Family status**		
Married	315	75.4
Divorced	11	2.6
Widow	34	8.2
Single, never being married	58	13.8
**Diagnosis** (in month)		
<1	176	42.8
1-2	70	16.8
>2	172	41.1
**Type of cancer**		
Breast	57	13.7
Prostate	36	8.6
Bladder	15	3.6
Head and neck	23	5.5
Lung	57	13.6
Pancreatic	19	4.5
Liver	32	7.5
Melanoma	31	7.4
Cervical Cancer	47	11.3
Soft tissue cancer (non-melanoma)	25	6.1
Brain & spinal cord tumours	19	4.5
Other	57	13.6
**Type of treatment**		
Surgery	69	47.2
Radiotherapy	30	7.2
Chemotherapy	233	55.7
Surgical, chemotherapy and radiotherapy	42	10.1
Chemotherapy and radiotherapy	44	10.5
**Days of treatment**		
2-4	183	43.8
5-7	81	19.4
8-10	33	7.9
11-13	39	9.3
>13	82	19.6
**Received care for first time**		
Yes	226	54.1
No	192	45.9
**Received previous care**		
Yes	294	70.3
No	124	29.7
**Educational level**		
Primary Level of Education (Elementary School)	124	29.6
Secondary Level of Education (High School)	105	25.1
Undergraduate Education (University Graduate)	150	35.8
Post-Graduate Education (Master Level)	20	4.8
PhD level of education (Doctoral studies)	5	1.2
No Formal education	14	3.3

### Content validity

As discussed earlier, content validity for the item sets was addressed by grounding the questionnaire development in earlier survey instruments, by using a conceptual framework for quality nursing care and by developing the scale with guidance from an experts’ panel.

The calculated content validity ratio (CVR) for the 34 items of the QONCS varied from 0.73 to 1. Items 20, 21 and 23 had the lowest level of agreement between the experts (CVR = 0.73) and items 4, 5, 6, 11, 18, 22, 26 and 30 had the highest (CVR = 1) indicating that there was perfect agreement between the experts (CVR = 1). A satisfactory level of Content Validity Index (CVI) for the QONCS was found (CVI = 0.84) among experts suggesting that the scale had a good content validity [26].

### Instrument structure

During several steps, a total of eleven items from the initial version of the scale were eliminated because they did not contribute to a simple factor structure and failed to meet a minimum criteria of having a primary factor loading of 0.35 or above. The 34 items of the final QONCS had an after rotation primary factor loading of 0.36 or above and only in four occasions, cross-loading of 0.23 or above (Table 3). Kaiser-Meyer-Olkin coefficient for sampling adequacy was estimated 0.913 while for the Bartlett test of sphericity x2 = (12506.097) df = 666, p < =0.001, suggesting that the data were appropriate to be subjected to factor analysis. The diagonals of the anti-image correlation matrix were all over 0.5, supporting the inclusion of each item in the factor analysis. Finally, the communalities were all above 0.4 further confirming that each item shared some common variance with other items. Given these overall indicators, factor analysis was conducted with all 34 items.

**Table 3:** **Factor loadings of the exploratory factor analysis results for quality of oncology Nursing care scale – after rotation** ^**a**^ **results**

	**Being supported and confirmed**	**Spiritual caring**	**Sense of belonging**	**Being respected**	**Being valued**	**Communalities**
Nurse is emotionally supportive	0,944	0,012	0,089	−0,062	−0,193	0,80
Nurse strives to establish good communication with patients	0,902	0,001	0,011	−0,042	−0,029	0,83
Nurse communicates well during the care with patients	0,871	0,015	−0,034	−0,055	−0,036	0,75
Patients can rely on a nurse	0,822	0,037	0,132	0,021	0,01	0,79
Nurse responses promptly to questions and concerns	0,698	0,031	0,046	−0,034	0,119	0,70
Nurse express a real interest	0,655	0,018	0,054	−0,046	0,139	0,66
Nurse’s actions gain trust	0,594	0,072	0,049	−0,006	0,234	0,67
Impression of being in good hands	0,499	0,032	0,004	−0,033	0,384	0,71
Nurse is competent in relation to equipment and technology	0,495	−0,063	0,26	0,074	0,251	0,62
The nurse acknowledges the caring needs	0,494	0,277	−0,04	0,038	−0,16	0,69
Nurse is knowledgeable in relation to patients condition	0,469	0,004	0,072	0,032	0,41	0,69
The nurse provides info in a comprehensive way	0,457	0,073	−0,168	−0,251	0,249	0,62
The nurse respects the needs and provides information	0,411	0,002	−0,041	−0,218	0,367	0,71
Patients receive the care of their choice	0,392	0,102	0,019	−0,205	0,257	0,46
The nurse answers the questions honestly	0,363	0,006	−0,035	−0,318	0,289	0,66
Feeling of asking nurse anything	−0,384	−0,009	−0,065	−0,184	0,127	0,58
Nurse is interested to know patients’ view on life and death	−0,048	0,879	−0,049	0,028	−0,001	0,75
Nurse initiates discussion around spiritual issues	−0,023	0,865	−0,125	−0,018	−0,085	0,73
Nurses availability to discuss/encourage spiritual issues	0,032	0,821	−0,043	0,002	0,035	0,68
Nurse is interested in clarifying the religious preferences	−0,056	0,762	−0,012	0,045	−0,012	0,57
Nurse sensitiveness and respect towards the religious preferences	0,079	0,588	0,143	−0,025	0,016	0,44
Nurse facilitates the religious rituals while receiving care	0,05	0,569	0,156	0,004	0,049	0,41
Nurse clarifies the desire of family presence	0,132	−0,048	0,839	0,048	0,023	0,78
Nurse acknowledges the importance of family’s presence	0,041	−0,099	0,762	−0,008	0,194	0,77
Nurse encourage family participate in decision-making	−0,005	0,009	0,741	−0,144	0,022	0,67
Nurse encourages the presence of family during care	0,14	0,087	0,732	0,049	0,024	0,65
Nurse involves family in the delivery of the care	−0,11	0,089	0,695	−0,16	−0,033	0,53
Option to participate in the decision-making regarding the n/c	−0,072	−0,024	0,103	−0,944	−0,048	0,85
Nurse provides adequate information in order to participate in d/m	0,039	−0,05	0,138	−0,828	0,012	0,84
The nurse cares with respect	0,268	0,074	−0,023	−0,542	0,146	0,69
Being cared for adequately by the nurses	0,014	−0,012	0,011	0,000	0,865	0,77
Patients receive care that condition calls upon	−0,052	−0,006	0,144	−0,008	0,834	0,76
The nurse is caring and understanding	−0,003	0,037	0,044	−0,038	0,82	0,74
The nurse is caring in a compassionate way	0,012	0,015	0,06	−0,086	0,767	0,73

Extraction Method: Maximum Likelihood.

Rotation Method: Oblimin with Kaiser Normalization.

^a^Rotation converged in 9 iterations.

The exploratory factor analysis of the remaining 34 items, using Maximum Likelihood method with Oblimin rotation method with Kaiser Normalization to account for the relationship among the factors, yielded a five-factor structure that explained 68.53% of the variance of the data (Table 3). Factor 1 consisted of 16 items: “being supported and confirmed” and explained 45,81% of the total variance; factor 2 consisted of 6 items: “spiritual caring” and explained 9,93% of the variance; factor 3 consisted of 5 items: “sense of belonging” and explained 6,27% of the total variance; factor 4 consisted of 4 items: “being valued” and explained 2,82% of the total variance and factor 5 consisted of 3 items: “being respected” and explained 3,69% of the total variance. Cross-loaded items were inspected in terms of content and rationally categorised into the factor that was most relevant. The 3 items, “impression of being in good hands”, “nurse is knowledgeable in relation to patients condition”, “the nurse respects the needs and provides information” loaded, in a reasonable fashion, on “Being Supportive” and “Being Valued” factors and the authors decided to fit them in the “Being Supportive” factor (Table 3). The item “the nurse answers the questions honestly” loaded on both the “Being Supportive” (0.363) and the “Being Respected” (−0.318) and was fitted to the former as well.

### Test-retest reliability

Internal reliability was confirmed for each of the subscales with Cronbach’s alpha being 0.83 for factor 1, 0.90 for factor 2, 0.90 for factor 3, 0.91 for factor 4 and 0.86 for factor 5. Correlation coefficient between test and retest for the overall scale was r = 0.79. This result indicates that the scale had adequate stability reliability.

## Discussion

The aim of this paper was to describe the rigorous process for the development and psychometric testing of a cancer specific questionnaire designed to explore the quality of the provided nursing within the oncological setting. The complex care demanded in these settings as well as the need for advanced needs assessment calls for cancer specific instruments that can perform well within these requirements as a means to improve the provided cancer care. The results suggest that the QONCS is a consistent (excellent internal consistency and adequate stability reliability) and valid instrument to provide an assessment of the provided quality of oncology nursing care.

The QONCS has some noteworthy advantages relative to previous questionnaires of quality nursing care. From a conceptual point of view, this is the first instrument that attempts to cover the main domains of quality nursing care as these were described by patients, nurses and patients advocates in a previous qualitative study [1,20] as well as research in the area [27,28].

Compared to the preceding questionnaires including the OPPQNCS, only the QONCS adopts a holistic approach to assessing the patients’ needs within the oncological setting. As part of this approach it attributes emphasis to an important area of the care for patients diagnosed with cancer, that of spirituality, an aspect that remained unexplored and unaddressed by other similar questionnaires [29-31]. Adding to its properties, this instrument was validated in patients diagnosed with a wide range of cancer types and at different phases of the cancer journey, making it appropriate for use in these groups.

The QONCS is a relatively short scale organized into five subscales that correspond to cancer patients’ perceptions of quality nursing care as these were identified through a rigorous process and informed by a conceptual framework. The items of the instrument are scored on a Likert-type scale, with a higher score indicating more self-perceived quality of nursing care, greater self-perception of support, value, confirmation and respect during the care, facilitation of belonging and more attention attributed to the spiritual needs of the patient during the care.

The scale adopts a multi perspective of the topic since its content derived from patients’ perspectives, the relevant literature, and professional experts. These procedures, complemented with theoretical definition of the constructs covered by the instrument, and with experts review over items, contributed to support its content and face validity.

The factor analysis (Maximum Likelihood) identified the domains of quality nursing care that derived from the hermeneutic phenomenological study (conceptual framework) and the review of the relevant literature as part of designing the questionnaire. This means that the patients discriminated between the distinct elements of nursing care.

### Limitations

Although this study successfully developed a new quality of nursing care scale within the oncological setting and subsequently validated this instrument, the results should be read in light of some limitations. The instrument was developed and validated in Cyprus and Greece, therefore its cultural origins and influences need to be acknowledged before applying it to other cultural specific settings. However, this is a common problem acknowledged for instruments developed in non English-speaking populations. Overcoming this problem that will increase the instrument’s transferability to other populations and cultural backgrounds, the process of translation, cultural adaptation and validation needs to be implemented. This process should follow internationally accepted procedures to ensure the resulting new language versions of the questionnaire are not only valid but also culturally specific. One further limitation is that the responsiveness of the QONCS was not evaluated as part of this study nor the criterion validity. In relation to the latter, as there is only one other questionnaire that measures quality nursing care in this group of patients but fails to integrate aspects such as spiritual care we believe that it was not appropriate to be considered as a gold standard questionnaire. Finally, the study excluded very ill patients at the end of the cancer trajectory. Further validation is warranted before the instrument can be recommended for use with this particular group of patients. Despites its limitations, the new instrument has been validated through a rigorous process, whilst the validation study included various cancer patients’ populations and various research sites in two European countries.

## Conclusions

Valid and reliable questionnaires of the self-perceived quality nursing care provided within the oncological setting are essential if the data are to be used in quality improvement. The rigorous development process has yielded a 34-item questionnaire that can capture the specificities of the nursing care provided to patients diagnosed with cancer and receive care in the hospital. The present study has provided strong evidence for the internal consistency, stability reliability, content and construct validity of this questionnaire. The QONCS is recommended for future applications designed to assess the self-perceived quality of nursing care provided to patients with cancer receiving care in oncological settings in Cyprus and Greece and similar settings in other countries. As with any new questionnaire, its use in different settings will accumulate more robust evidence about its construct validity.

Given the limited number of appropriate questionnaires for evaluating quality nursing care in patients with cancer receiving hospital care, these results are promising. As this is the first exploration of the QONCS, further research is warranted to examine the factor complexity in larger and more diverse samples, provide evidence based on relations with other variables including both convergent and divergent evidence, and explore confirmatory factor analyses.
